# Multifactorial Analysis of Environmental Metabolomic Data in Ecotoxicology: Wild Marine Mussel Exposed to WWTP Effluent as a Case Study

**DOI:** 10.3390/metabo10070269

**Published:** 2020-06-29

**Authors:** Thibaut Dumas, Julien Boccard, Elena Gomez, Hélène Fenet, Frédérique Courant

**Affiliations:** 1 HydroSciences Montpellier, University of Montpellier, CNRS, IRD, 34093 Montpellier, France; maria-elena.gomez-hernandez@umontpellier.fr (E.G.); helene.fenet@umontpellier.fr (H.F.); frederique.courant@umontpellier.fr (F.C.); 2 School of Pharmaceutical Sciences, University of Geneva, 1211 Geneva, Switzerland; julien.boccard@unige.ch; 3 Institute of Pharmaceutical Sciences of Western Switzerland, University of Geneva, 1211 Geneva, Switzerland

**Keywords:** gender-specific response, non-targeted metabolomics, environmental metabolomics, liquid chromatography-high resolution mass spectrometry, wastewater treatment plant effluent, *Mytilus galloprovincialis*, bivalve mollusks, multifactor experimental design, analysis of variance multiblock orthogonal partial least squares

## Abstract

Environmental metabolomics is a powerful approach to investigate the response of organisms to contaminant exposure at a molecular scale. However, metabolomic responses to realistic environmental conditions can be hindered by factors intrinsic to the environment and the organism. Hence, a well-designed experimental exposure associated with adequate statistical analysis could be helpful to better characterize and relate the observed variability to its different origins. In the current study, we applied a multifactorial experiment combined to Analysis of variance Multiblock Orthogonal Partial Least Squares (AMOPLS), to assess the metabolic response of wild marine mussels, *Mytilus galloprovincialis*, exposed to a wastewater treatment plant effluent, considering gender as an experimental factor. First, the total observed variability was decomposed to highlight the contribution of each effect related to the experimental factors. Both the exposure and the interaction gender × exposure had a statistically significant impact on the observed metabolic alteration. Then, these metabolic patterns were further characterized by analyzing the individual variable contributions to each effect. A main change in glycerophospholipid levels was highlighted in both males and females as a common response, possibly caused by oxidative stress, which could lead to reproductive disorders, whereas metabolic alterations in some polar lipids and kynurenine pathway were rather gender-specific. This may indicate a disturbance in the energy metabolism and immune system only in males. Finally, AMOPLS is a useful tool facilitating the interpretation of complex metabolomic data and is expected to have a broad application in the field of ecotoxicology.

## 1. Introduction

Coastal areas around the world are subjected to multiple pressures, including an increase of submarine sewage outfalls. Conventional wastewater treatment plants (WWTPs) continuously release a wide diversity of contaminants in aquatic environments [1,2,3]. Even at low environmental concentrations (ng–µg/L), those contaminants pose a threat to marine organisms, especially sessile ones [4,5,6,7,8]. As little is known about how WWTP effluents affect those organisms, investigation at a molecular level could be useful to predict the effect at a higher level of biological organization. Approaches helping to characterize the response of marine organisms to such multi-contamination without preconception are key for understanding the impact of WWTP effluents on coastal areas.

Omics approaches now make it possible to obtain considerably more complete and specific information on the biochemical response of organisms to a stress. More recently, environmental metabolomics has been commonly and efficiently applied to investigate more deeply mechanisms and modes of action of single contaminants or mixtures [9,10,11]. This approach is based on the identification of low molecular weight metabolites (50–1500 Da) whose production and levels vary with the physiological, developmental, or pathological state of cells, tissues, organs, or whole organisms [10,12]. It must be noted that mass spectrometry-based metabolomics is a holistic approach and provides a huge amount of data that reflects also metabolic changes related to factors intrinsic to the environment (i.e., temperature, pH, predation, food availability, etc.) and the organism (i.e., gender, age, size, genetic, etc.). Hence, it can be difficult to unravel the response of wild organisms to contaminant exposure that can be mixed with variations generated from those factors [13]. In this way, integrating multiple factors to an adapted experimental design could be an efficient methodology to better understand the role of those factors in the response of wild organisms to contaminants by being closer to the reality of complex phenomena.

Gender has long been considered to be a confounding factor in response to environmental stressors and, as such, was often excluded from experimental design because of the time, cost, and difficulty of its determination [14,15]. However, gender can be a factor of primary importance since it plays a role in the genetics, physiology, morphology, and behavior of organisms and thus influences the uptake, fate, and toxicity of contaminants in organisms [14]. In recent decades, the awareness of gender as an experimental factor has increased with the emphasis on endocrine active chemicals, underscoring biochemical and physiological differences between the sexes [16]. Currently, omics approaches fully contribute to highlighting the gender-specific response to contaminant exposure [15,17], including in invertebrates. Indeed, Ji et al. [18] observed a gender-specific metabolic response of mussel *Mytilus galloprovincialis* to the flame retardant BDE 47. Impacted metabolites in male mussels (e.g., threonine, glutamate, lysine, and histidine) suggest a change in energy metabolism, while a disturbance of both osmoregulation and energy metabolism is rather demonstrated in females (modulation of the osmolytes betaine and hypotaurine, ATP elevation). Other studies in environmental metabolomics also demonstrated the gender-specific response of mussels exposed to the pesticide triazophos [19], the flame retardant tetrabromobisphenol A [20], bisphenol A [21], and the pharmaceutical active compound fadrozole hydrochloride [22], or challenged by pathogens [23,24]. However, those studies did not fully take advantage of the multifactorial experimental structure of the data to characterize both exposure effects and gender-specific responses and to relate the observed variability (metabolic changes) to the main factors or their interaction in a multivariate context.

One of the challenges in environmental metabolomics is to better characterize and relate the observed variability to its different origins in order to correctly interpret biological responses to environmental stressors [25]. This could be overcome thanks to well-designed experimental exposure associated with dedicated statistical analysis, as discussed in Simmons et al. [25]. However, when multiple influences come into play, it becomes challenging to relate metabolic alterations to specific factors. In that context, systematic strategies taking advantage of experimental design become crucial to plan the experiments and analyze the data generated in an adequate manner. Design of experiments aims to rationally structure the different levels (e.g., male/female or control/exposed) taken by the experimental factors (e.g., gender or exposure) to efficiently distinguish the different effects leading to specific variations in the measured data but occurring simultaneously. Dedicated statistical data analysis approaches are then required to account explicitly for the experimental setup and assess the respective effects and potential interactions between experimental factors. Analysis of variance (ANOVA) offers a classical statistical framework to separate the different sources of variability. However, its application becomes limited when a large number of signals are measured to characterize observations, as is the case in metabolomics. It should be noted that metabolomic data can be investigated using both univariate and multivariate techniques. While the former are based on standard one-variable-at-a-time analysis, they remain a classical solution to estimate the statistical merit of a given metabolite. Because the latter allow global metabolic patterns to be considered in the model, they have the capacity to uncover relationships between subsets of metabolites carrying common information, which could be overlooked otherwise. They are therefore especially well-suited to the analysis of untargeted metabolomic datasets. In this context, the combination of multivariate methods and experimental design theory has led to the development of new methods. Among the various solutions developed to address this problem, the combination of ANOVA decomposition and projection methods such as principal component analysis (PCA), simultaneous component analysis (SCA), or partial least squares (PLS) regression has been shown to be a relevant approach [26]. These tools allow the different trends associated with each effect to be efficiently summarized using a limited number of mutually orthogonal latent variables (or components). Recently, a method integrating ANOVA-based submatrices associated with each effect/interaction into a single multiblock orthogonal PLS model (ANOVA multiblock OPLS (AMOPLS) [27]) has been shown to be very effective in distinguishing multiple metabolic alterations and facilitating interpretation. In the same way as standard factorial models, interpretation can be done on the basis of score and loading plots to assess the distribution of observations and the contributions of the variables, respectively. As all experimental effects are integrated into a single multiblock model, this technique provides an objective evaluation of multiple factors potentially influencing metabolites in a concurrent manner. The computation of distinct variable importance in the projection (VIP) values related separately to each effect provides a relevant strategy to decompose the contribution of the variables to the model. Finally, the statistical significance of each effect can be empirically estimated using permutation tests.

In a previous study (Dumas et al. [28]), we demonstrated the deleterious metabolic effects of a WWTP effluent extract in digestive glands of male *Mytilus galloprovincialis* mussels. Here, we propose to apply the AMOPLS method for the first time in environmental metabolomics in order to (i) bring out metabolites modulated in the same way in male and female mussels (i.e., gender-independent response caused by the WWTP effluent exposure), and (ii) gain insight into how gender influences responses to exposure (qualitatively and quantitatively). The experimental design was conducted in controlled laboratory conditions to simplify environmental exposure, limiting as much as possible the variation generated by confounding factors, and to better characterize the response of marine mussels to WWTP effluents. Finally, we further discuss the new perspectives offered by AMOPLS for the analysis of environmental metabolomics in the field of ecotoxicology.

## 2. Results and Discussion

### 2.1. General Overview of the Dataset

LC-HRMS analysis of digestive glands collected from mussels exposed either to solvent control (SC, *n* = 9 males and *n* = 10 females) or WWTP effluent extract (*n* = 10 males and *n* = 10 females) was carried out in both ESI^+^ and ESI^−^ modes. Metabolic fingerprints composed of 3338 features detected in ESI^−^ and 1460 features in ESI^+^ were obtained after cleaning the datasets, as described in Section 3.6.1. Analytical repeatability was acceptable as the percentages of features with as RSD < 30% in ESI^−^ and ESI^+^ were 68% and 65%, respectively.

### 2.2. Contribution of Experimental Factors to the Total Variability

The experimental design involved two factors, namely *Exposure* (two levels: SC or WWTP exposure) and *Gender* (two levels: male or female). The main effects contributions to the total observed variability of the *Gender* factor, *Exposure* factor, as well as their interaction *Gender × Exposure*, were evaluated using the AMOPLS approach (Table 1).

The *Gender* main effect (differences between male and female on average) contributed only to a small fraction of the total variability (3.6%). This contribution was deemed statistically non-significant based on permutation tests (*p* > 0.05), leading to the hypothesis that differences between males and females on average (i.e., independently of *Exposure* factor) could be considered negligible. On the other hand, the *Exposure* main effect (WWTP vs. SC) accounted for 7.5% of the total variability and was found to be highly significant (*p* < 0.01), demonstrating that WWTP effluent extract had an impact on the metabolism of mussels whatever the gender.

The *Gender × Exposure* interaction, related to the gender-specific response to WWTP effluent extract, explained 3.9% of the total variation and was also found to be significant (*p* < 0.05). Although differences between genders (on average) did not cause a significant impact on metabolomic patterns, this result suggests that mussels may respond differently to the exposure according to gender. 

The residuals constituted the remaining 85.0% of the total observed variability that could not be related to the experimental factors. This unexplained part therefore accounts for most of the variation observed in the dataset, showing that other sources influenced the variability of the metabolomic profiles, whether analytical or biological. Although individuals were collected at the same location of the mussel culture, they were still wild organisms with potentially different life history traits and/or a different physiological status, thus leading to important variations in their metabolism and response to WWTP effluent extract exposure.

An AMOPLS model with three predictive (tp) and one orthogonal (to) components was found optimal using permutation tests (R^2^ = 0.92, *p* < 0.01). Model interpretation was then based on the predictive components associated with *Exposure* (tp1), *Gender × Exposure* (tp2), and *Gender* (tp3), as illustrated by the block contributions summarized in Table 1. The orthogonal component related to the unexplained part of variability, i.e., residuals, was not further investigated. VIP^2^ values were computed for all variables to rank ion features according to their importance for each specific predictive component. By these means, subsets of metabolite candidates potentially associated with each effect could be highlighted.

### 2.3. Impact of Exposure

**Figure 1:**
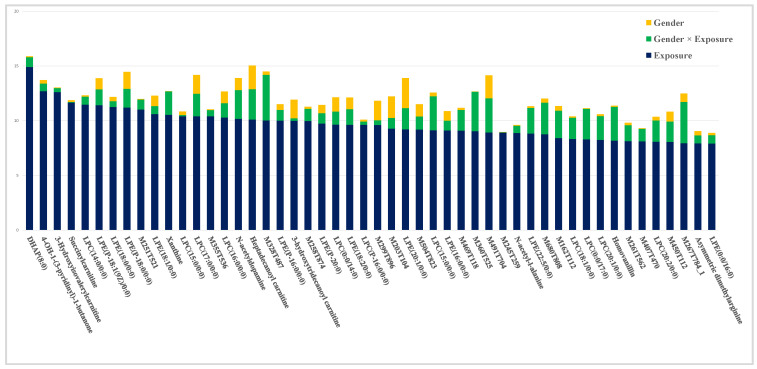
Effect-specific variable importance in the projection (VIP^2^) values for the 50 highest ranked metabolites according to the impact of the *Exposure* factor.

**Figure 2:**
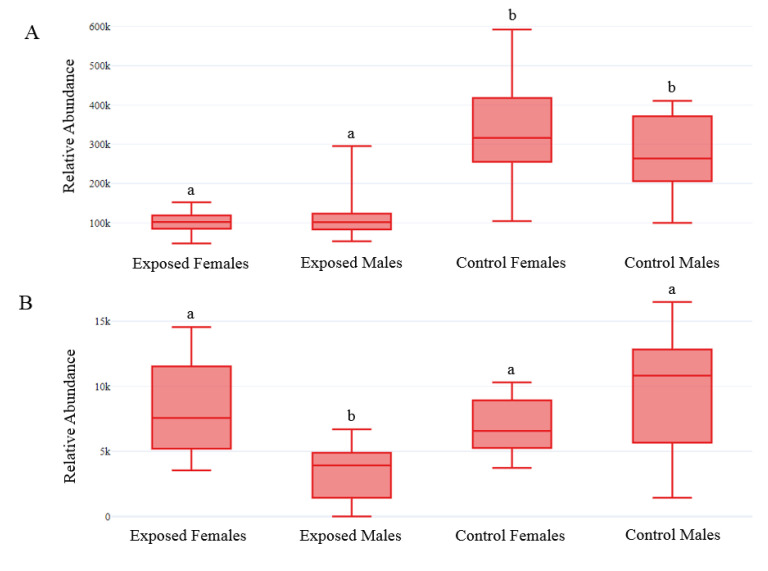
Boxplots illustrating an exposure-specific modulation of LPC (14:0/0:0) in both male and female mussels (**A**) and a gender-specific modulation of 1-pentadecanoyl-glycero-3-phosphate only in males (**B**). Different letters (a and b) mean significant difference (*q*-value < 0.05) between relative abundances.

The top 50 values of effect-specific VIP^2^ related to *Exposure* were selected to find the most relevant metabolites contributing to differences between WWTP and solvent control (SC) exposed groups (Figure 1). Then, relative abundances of these metabolites were compared across the different samples, to determine whether they were down or up-modulated in response to WWTP exposure (Supplementary Materials Table S1). Most of them followed the same pattern and were significantly down-modulated in both male and female exposed organisms, as illustrated with the boxplot of lysophosphocholine (14:0/0:0) (Figure 2A). Interestingly, the similar modulations observed in the two genders validates the relevance of results provided by tp1 for exposure. Indeed, tp1 aims to highlight metabolites whose modulation is explained by the *Exposure* factor, independently of others factors such as gender.

As reported in Table S1 in the Supplementary Materials, most of the metabolites were annotated at level 2 (*n* = 31), while others remained still unknown (level 4; *n* = 17) or have been confirmed (level 1; *n* = 2). This raised the difficulty of identifying these metabolites and the lack of available information concerning the metabolism of bivalve mollusks. However, a great majority seemed to be part of polar lipid metabolism, belonging to the class of glycerophospholipids such as lysophosphatidylcholines (LPCs) and lysophosphatidylethanolamines (LPEs), and also acyl carnitines and aryl alkyl ketones compounds. Some of those LPCs (e.g., 14:0/0:0; 15:0/0:0; 17:0/0:0; 18:1/0:0; 20:1/0:0) and LPEs (e.g., P-16:0/0:0; P-18:1(9Z)/0:0; P-18:0/0:0; 18:0/0:0) have already been identified in *M. galloprovincialis*, which reinforces these annotation hypotheses [29,30].

Changes in polar lipids underlined by the AMOPLS model may indicate a crucial role of lipids in response to WWTP effluent exposure in both male and female mussels. In bivalves, lipids exert several roles. They are important structural components of biological membranes, energy reserves, precursors of secondary messengers and transcription factors, act in reproduction and sexual maturation, and are active in immunological response and environmental adaptation [31]. More specifically, glycerophospholipids such as glycerophosphocholines (PCs) and glycerophosphoethanolamines (PEs), are the main lipid components of biological membranes and ensure the required fluidity of the lipid bilayer for the normal functioning of membrane bound proteins, ion channels, and receptors [32]. In our study, most of the impacted metabolites belong to the class of glycerophospholipids. LPC and LPE, derived from PC and PE respectively, correspond to the loss of an acyl chain, resulting in the release of fatty acid from the sn-2 or sn-1 position, caused respectively by the phospholipase A2 (PLA2) or A1 activity [33]. Hence, this gain in polarity made possible the observation of LPC and LPE, consistence with our extraction and analysis method, while PC and PE may also be disturbed but are less polar and cannot be observed in this study. However, little is known about the role of LPC and LPE in mussels. Partly present in biological membranes, they may increase membrane polarity, leading to a decrease in membrane fluidity and permeability, or be involved in reactions with amino compounds and DNA bases, interfering with the functional integrity of the cell membranes [34].

Although several sources may vary the lipid content and composition in mussels (i.e., food availability, temperature, salinity, animal life cycle, sex, and spawning), changes in lipid metabolism are also known to be a biochemical response to pollutant exposure in *Mytilus* spp. [35,36,37,38,39]. Recently, Cappello et al. [36] observed a depletion of glycerophospholipids in *M. galloprovincialis* after a 7-day exposure to the pharmaceutical active compound drospirenone (10 µg/L). This may be suggestive of oxidative stress, since PCs are one of the degraded components of biological membranes during lipid peroxidation. Oxidative stress induced by this WWTP effluent exposure can also be supported with an increase of oxidized glutathione in males (trend, *q*-value = 0.095) and also in females (significant, *q*-value = 0.024) (results not shown), that may explain in this study the alteration of glycerophospholipids. Furthermore, LPCs are recognized to activate multiple signaling pathways, through the G protein-coupled receptor, involved in oxidative stress and inflammatory responses [40]. As mentioned above, hydrolysis of PC by PLA2 produces LPC and free fatty acid. The latter is frequently arachidonic acid, the precursor of the eicosanoid family of potent inflammatory mediators including prostaglandins, thromboxanes, and leukotrienes [41]. Worthy of note, eicosanoids are thought to play a role in the reproductive physiology of bivalve mollusks during both gametogenesis and spawning [42], which corroborates a potential ecotoxicity on these organisms.

According to the crucial role of lipids in response to WWTP effluent exposure in both male and female mussels, a lipidomics study would be suitable for a better understanding on the impact of lipid metabolism and consequences on the organisms, since they are involved in essential functions.

In this study, the first aim was to bring out a common response caused by WWTP effluent exposure in wild marine mussels collected directly from the environment, which were homogenous in appearance (i.e., same species and same shell size) but finally physiologically heterogeneous (i.e., difference of gender and probably other parameters not measured, such as stage of sexual maturation, age, etc.). Despite an important biological variability, the AMOPLS method allowed the proportionally small part of meaningful information to be efficiently extracted. In this way, a common response to exposure was highlighted in mussels, independently of gender or other physiological differences. Hence, the predictive component related to *Exposure* could be helpful in the case of biomarker discovery, as it can serve as a basis to highlight the metabolites that respond the most to exposure and not to confounding factors that can bias the interpretation of the observed variation.

### 2.4. Impact of the Interaction Exposure × Gender

**Figure 3:**
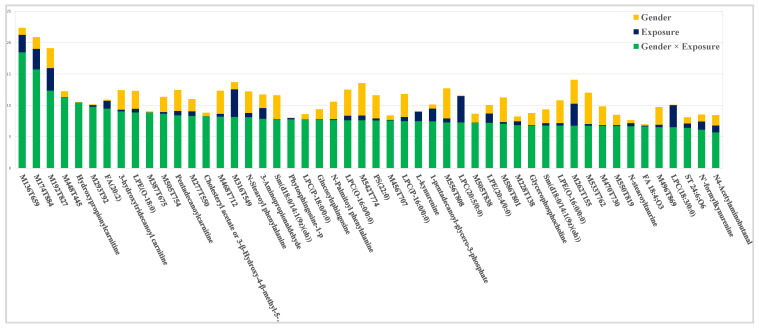
Effect-specific variable importance in the projection (VIP^2^) values for the 50 highest ranked metabolites according to the impact of the interaction *Gender × Exposure*.

The AMOPLS model also revealed a significant effect of the interaction between the two factors *Exposure* and *Gender*. To facilitate interpretation, effect-specific VIP^2^ computed for tp2 allowed us to highlight the most important metabolites influenced by the gender-specific response to WWTP effluent exposure (Figure 3). Interestingly, this variable subset included metabolites modulated in an opposite way between male and female mussels or only in one sex (Supplementary Materials Table S1). An example of a gender-specific response is illustrated in Figure 2B. However, the pairwise multiple comparison of those modulations indicated only slightly significant differences when exposed males or females were compared to their respective controls. Nevertheless, other statistical comparisons given in Supplementary Materials Table S2 showed significant differences between exposed males and females, while no differences were observed between controls. Hence, the gender-specific response could be observed in different ways, depending of the group comparisons and the direction of modulation.

As in the case of the *Exposure* effect, a majority of gender-specific impacted metabolites were annotated (level 2) as polar lipids belonging to the class of glycerophospholipids (i.e., LPC, LPE, and phosphatidylserines), sphingolipids and glycosphingolipids, sterol lipids, acyl carnitines, fatty amides, and also fatty acyls compounds. A general trend showed an up-modulation of those compounds in male mussels, while a down-modulation was rather observed in females. Although the glycerophospholipids class was impacted in both male and female mussels, the modulation of some LPCs and LPEs seemed to be gender-specific. Due to the lack of information about many of these metabolites in mussels, the interpretation of the gender-specific response remained quite limited. However, differences in phospholipid and triacylglycerid content between male and female *M. galloprovincialis* mussels have already been related to the gametogenic cycle [43]. They play important roles in the reproductive process. In females, triacylglycerides are very important energy reserves in the oocytes, assuring the viability of larvae, while male spermatozoa do not accumulate triacylglycerid reserves, but lipids mainly incorporate into membranes as phospholipids. These differences of lipid contents and use indicate intrinsic biological differences between male and female mussels. This could therefore explain partially the gender-specific responses of *M. galloprovincialis* to WWTP effluent exposure.

A down-modulation of l-kynurenine and N’-formylkynurenine involved in the gender-specific response to WWTP effluent exposure could also be highlighted based on the AMOPLS model. Interestingly, thanks to the experimental setup implemented in this study, this alteration of the kynurenine pathway was shown to be specific to male mussels. In humans, the kynurenine pathway has received increasing attention because of its connection to the immune system and the de novo synthesis of nicotinamide adenine dinucleotide (NAD+), which plays a critical role in many enzymatic redox reactions and in mitochondrial energy production [44]. A potential toxicity of the WWTP effluent on the immune system and energy metabolism on male mussels was also demonstrated in our previous study [28]. However, further studies are needed to confirm the role of the kynurenine pathway in marine mussels.

The second aim of this study was to determine quantitatively and qualitatively how gender influences the response of marine mussels to WWTP effluent exposure. Despite the fact that metabolic alterations associated with this effect represented less than 4% of the total variability, the AMOPLS method revealed a significant impact of the interaction *Gender × Exposure*, and highlighted specific metabolites modulated either in males or in females, or both, but in opposite ways. Because of its limited magnitude, it is important to validate the relevance of these results biologically by linking the highlighted metabolites to physiological processes that may differ between male and female mussels. However, further studies are required to confirm the structural annotation of these metabolites (Supplementary Materials Table S1) and examine their role in mussel response to contaminants.

### 2.5. Perspective of Environmental Metabolomics in Ecotoxicology Based on Multifactorial Experiments

The complexity and heterogeneity of biological and environmental systems can influence the uptake, fate, and toxicity of contaminants in organisms. Hence, understanding these phenomena has become one of the main challenges in ecotoxicology. In the same way, this complexity has an amplified impact on environmental metabolomics, since the sensitivity of this approach, when performed with mass spectrometry, allows very subtle metabolic changes that are not always related to the studied factors to be monitored. To overcome this issue, dedicated chemometric tools accounting explicitly for the experimental factors, such as AMOPLS, can relate the observed variation to their main effects and interactions in a single model, and then facilitate the interpretation of the complex dataset obtained from multifactor experimental design. In this way, the AMOPLS method, taking advantage of experimental design, is expected to have a broad application in the field of ecotoxicology.

In this study, we demonstrated the ability of AMOPLS to bring out relevant information related to WWTP exposure despite the large part of unexplained variability, resulting probably from the biological diversity of wild marine mussels. As a consequence, AMOPLS could be a suitable screening step in biomarker discovery for environmental monitoring, as it can select specific metabolites related to exposure from a complex dataset. In another way, it could also be possible to reduce the unexplained variability (i.e., residuals) by accounting for additional physiological factors in the analysis (e.g., age, stage of sexual maturity, etc.). In addition, this method, combined with environmental metabolomics, could provide new insights into the influence of physiological parameters of organisms in response to contaminants.

It has to be noted that AMOPLS can be applied to any fixed-effects multifactorial experimental design. For instance, both dose and time factors are very important in ecotoxicology as they define contaminant toxicity. González-Ruiz et al. [45] already asserted the efficiency of the method for such a structure of experimental design in human toxicology. They demonstrated a dose effect (0, 0.5, and 1 µM) and a time effect (24 h and 10 days) of trimethyltin on neural cells, thanks to a simplified interpretation of metabolite variations. Another application of AMOPLS could be the use of experimental design integrating environmental factors (e.g., changes in temperature, salinity, pH, etc.), that can influence the uptake, fate, and toxicity of contaminants in organisms. These kinds of experimental designs are largely widespread in the field of ecotoxicology in the context of climate change. Moreover, multifactorial experiments involving different biological layers, i.e., multi-omics approaches, constitute a very promising area of research to offer a more integrative view of the impact of contaminant exposure on sentinel species.

By gradually integrating the complexity and heterogeneity of organisms and environments into the experimental design, the shift of environmental metabolomics studies from laboratory to the field would be more feasible. However, some limitations may be encountered. The addition of supplementary factors to the experimental design requires balanced groups with a sufficient number of individuals to be maintained; although the analysis of unbalanced designs is possible, more sophisticated approaches to variance decomposition are needed in that case [27].

It must be kept in mind that the proposed applications in ecotoxicology are not exhaustive and not limited to this field of research. Because it can be applied to all kinds of data generated from an experimental design, such methods are expected to have a broad field of application.

## 3. Materials and Methods

### 3.1. Chemicals

Pesticide analytical-grade solvents (methanol, dichloromethane, and ethanol) and LC/MS grade solvents (water, acetonitrile, formic acid 99%) were obtained from Carlo Erba (Val de Reuil, France). Ultrapure water was generated by a Simplicity UV system from Millipore (Bedford, MA, USA) with a specific resistance of 18.2 MΩ.cm at 25 °C. Analytically pure standards used for identification at level one [46] were purchased from the following suppliers: Sigma-Aldrich (now part of Merck), Santa Cruz Biotechnology, Toronto Research Chemicals, and LGC Standards.

### 3.2. WWTP Effluent Extract Preparation

The WWTP effluent preparation was followed by a Solid Phase Extraction (SPE), previously detailed by Dumas et al. [28]. Briefly, effluent (15 L) was first filtered through GF/C filters (1.2 µm). The conditioning of cartridges (Oasis HLB 500 mg, 6 cc) was carried out first with 5 mL of methanol and then 5 mL of distilled water. Afterwards, the WWTP effluent sample (500 mL) was passed through 30 cartridges under vacuum. After washing with 5 mL of Milli Q water, the cartridges were dried. Then, elution of the cartridges was carried out with 2 × 5 mL of methanol. Finally, the eluate of all cartridges was evaporated in order to obtain 3 mL of concentrated extract.

The solution used to spike glass aquaria during the exposure period was prepared extemporaneously every day. In a glass tube, 20 µL of concentrated extract was added to 80 µL of methanol. A final WWTP effluent extract dilution of 5% was reached in the aquarium by spiking the seawater (2 L) with 100 µL of the solution.

### 3.3. Animals and Experimental Design

The detailed experimental design was described by Dumas et al. [28]. Briefly, 50 *Mytilus galloprovincialis* mussels (5.3–7.5 cm shell length), purchased from a Mediterranean Sea mussel culture (Bouzigues, France), were distributed into different glass aquaria (2 L filtered seawater) with 5 individuals per aquarium and 5 aquarium per treatment (25 individuals per treatment). Seawater was kept under continuous aeration and renewed daily (static renewal), and physicochemical parameters (temperature, pH, salinity, and oxygen concentration) were checked throughout the experiment. Following a 7-day acclimatization period, mussels underwent two different treatments: either exposure to the solvent control (SC, 100 µL absolute methanol) or exposure to the WWTP solution (cf. Section 2.2). After the 7-day exposure period (no dead mussels), individuals were dissected for digestive gland collection (frozen at −80 °C) and sex microscopy determination. This work was conducted on 19 males (SC exposure *n* = 9 and WWTP effluent exposure *n* = 10) and 20 females (SC exposure *n* = 10 and WWTP effluent exposure *n* = 10).

### 3.4. Tissue Sample Preparation

The solvent system of methanol/dichloromethane/water (16/16/13; *v*/*v*/*v*) was used to extract metabolites from digestive glands of male and female mussels, as presented in previous works [28,47]. Briefly, the tissues (30 mg dry weight ± 0.25 mg) were homogenized and extracted in a first step with 240 µL of methanol and 120 µL of water and vortexed for 60 s. In a second step, tissues were then extracted with 240 µL of dichloromethane and 120 µL of water and vortexed for 60 s. Following a 15 min rest at 4 °C, the samples were vortexed again and then centrifuged for 15 min at 2000× *g* and 4 °C. The methanol/water phase (50 µL) was transferred to a glass tube and dried under a nitrogen stream. The extracts were re-suspended in 200 µL of acetonitrile/water (5/95; *v*/*v*). Afterwards, the extracts were filtered using a 0.20 μm PTFE syringe filter (Minisart SRP 4, Sartorius, Stonehouse, England) into vials prior to liquid chromatography—high resolution mass spectrometry (LC-HRMS) analysis.

### 3.5. Metabolic Fingerprint LC-HRMS Analysis

The metabolic fingerprints were generated on an Exactive Orbitrap HRMS (Thermo Fisher Scientific, Bremen, Germany) coupled to an LC system (Accela 600 pump, Thermo Fisher Scientific, Bremen, Germany). A reversed phase pentafluorophenylpropyl (PFPP) analytical column (100 × 2.1 mm; 3 μm particle size; Sigma Aldrich, PA, Bellefonte, USA) was used for the LC separation method. The injection volume of samples was 5 μL using full loop injection. The mobile phase was constituted of the two separate solvents water/acetonitrile both modified with 0.1% formic acid. The elution gradient (water/acetonitrile) was as follows: 95/5 from 0 to 3 min, 60/40 at 8 min, 50/50 at 9 min, 30/70 at 13 min, 5/95 from 15 to 18 min, and a re-equilibration period from 21 to 31 min at 95/5. The elution flow rate was fixed at 200 µL/min.

The HRMS was equipped with a heated electrospray ionization probe (HESI) source. Both ionization modes positive (ESI^+^) and negative (ESI^−^) were applied with the following parameters (ESI^+^/ESI^−^): spray voltage was set at |3.5|/|3.40| kV, capillary voltage at 45/−50 V, tube lens voltage at 90/−120 V, and skimmer voltage at 26/−25 V. The capillary and heater temperature was set at 250 °C. HRMS acquisitions were done in a full scan mode with a mass spectrum range of *m*/*z* 50–1000 at a mass resolution of 50,000 (FWHM, *m*/*z* 200).

In order to assess the analytical repeatability and sensitivity of acquisitions, a quality control (QC) sample was injected at regular intervals throughout the sequence. The QC sample was prepared by pooling 10 µL of each injected sample extract.

It has to be noted that a randomization procedure was set up during several experimental steps to avoid any bias and to reduce the influence of potential confounding factors. This includes the randomization of (i) mussel distribution in different glass aquaria before exposure, (ii) dissection, (iii) extraction, and (iv) LC–HRMS analysis.

### 3.6. Data Processing and Statistical Analysis

#### 3.6.1. Data Processing

Different steps for data processing were already presented by Dumas et al. [28]. Briefly, the raw data were converted into mzXML files with MSConvert freeware (ProteoWizard 3.0 [48]). A multi-step strategy was applied for processing ESI^+^ and ESI^−^ acquisitions separately using the XCMS package [49] in the R environment. XCMS returned results as a peak table containing variable identity (i.e., *m*/*z* and retention time) and feature abundances (i.e., peak area). After visual inspection of each extracted ion chromatogram, all features corresponding to baseline drift or background noise were discarded from the peak table. In addition, the Bioconductor package CAMERA [50] was used to remove isotopes, adducts, and fragments from the peak table, thus avoiding information redundancy. The analytical repeatability was assessed by calculating the relative standard deviation (RSD) for each features detected in QC samples. A controlled analytical variability was set such that 70% of features would have an RSD < 30% [51]. Features with an RSD > 30% were excluded for statistical analysis.

#### 3.6.2. Analysis of Variance Multiblock Orthogonal Partial Least Squares (AMOPLS)

The fusion of the data obtained from both ionization modes, i.e., ESI^+^ and ESI^−^, was carried out by concatenation after autoscaling and a normalization step based on the Frobenius norm of each dataset. AMOPLS was computed under the MATLAB^®^ 8 environment (The MathWorks, Natick, MA, USA). To avoid detrimental effects on interpretation due to unbalanced groups, a stratified subsampling strategy without replacement was carried out. Differences between glass aquaria were considered negligible, while the combination of gender and exposure experimental factors was used for the random selection of 9 mussels in each experimental group (i.e., the smallest size of exchangeable units). By these means, a population of 10^3^ balanced AMOPLS models was generated and parameters, including sum-of-squares, scores, and loadings, were then investigated to offer robust ensemble-based estimates. Additional details about this procedure can be found in Boccard et al. [52].

#### 3.6.3. Multivariate Metabolite Selection and Univariate Statistical Evaluation

Squared variable importance in the projection (VIP^2^) values were then calculated for both ESI^+^ and ESI^−^ modes. VIP^2^ values are considered to be the quantitative measure of the contribution of all modelled effects coming from a single variable [45]. By using the effect-specific VIP^2^, the total contributions to the model can easily be split into contributions through the different experimental factors, allowing the importance of each metabolite in separate significant effects to be evaluated (i.e., exposure, gender, and their interaction) [45]. 

Then, the 50 highest ranked metabolites according to their effect-specific VIP^2^ related to each factors were selected for annotation. Two-way ANOVA tests in conjunction with Tukey’s multiple comparison test were then performed on metabolite relative abundances with high effect-specific VIP^2^ in order to establish significant modulation. The false discovery rate was controlled using the Benjamini–Hochberg FDR correction [53]. Metabolites with a significant modulation higher than 30% (*q*-value < 0.05) were considered biologically relevant.

### 3.7. Metabolite Annotation and Identification

The annotation step was performed using the public databases Human Metabolome Database (http://www.hmdb.ca/) and LipidMaps (https://lipidmaps.org/). A mass precision was fixed at 0.001 Da. In line with the work of Sumner et al. [46], the levels of confidence for the annotation were defined as follows: (i) level 1 was characterized by unambiguous identification based on the accurate mass and retention time of the corresponding analytical standard injected under the same analytical conditions, and (ii) level 2 corresponded to putative annotation based upon physicochemical properties and/or spectral similarity with public databases.

## 4. Conclusions

Multifactor data analysis was demonstrated as a relevant method to characterize and relate the observed variability from structured metabolomic data to the studied factors. It provides an efficient quantification of the importance of their main effects and interactions, and highlights the contribution of metabolites with respect to each individual effect, all in a single model. This ability thus facilitates the interpretation of environmental metabolomic results, providing in our case meaningful information on the impact of WWTP effluent exposure in the marine mussel *Mytilus galloprovincialis*. Despite the high biological variability encountered in this study, a common response of both male and female mussels was first assessed with a main change in glycerophospholipid levels (e.g., LPC and LPE). This probably reflects an alteration of biological membranes caused by oxidative stress. In addition, LPC can also be related to the synthesis of arachidonic acid, a precursor of eicosanoids. The latter are involved in reproductive processes of mussels that may be disturbed by WWTP effluent exposure. As reproductive processes do not involve the same content and use of lipids between male and female mussels, a gender-specific response of several polar lipids was also highlighted. Furthermore, a specific modulation of the kynurenine pathway in male mussels may indicate a disturbance in energy metabolism and immune system, while these alterations were not observed in female mussels. In this way, our study also supports the importance of considering gender as an experimental factor. This current approach is expected to have broad application in the field of ecotoxicology.

## Figures and Tables

**Table 1:** Relative variability and block contributions of the AMOPLS analysis of metabolomic data acquired from digestive glands of male and female mussels (*Mytilus galloprovincialis*) exposed to WWTP effluent extract.

Factor	*RSS*	*p-Value*	Block Contributions			
			tp1	tp2	tp3	to
*Gender*	3.6%	>0.05	3.6%	7.3%	**84.1%**	25.5%
*Exposure*	7.5%	<0.01	**89.2%**	6.1%	4.9%	23.2%
*Gender × Exposure*	3.9%	<0.05	3.5%	**78.7%**	5.4%	25.1%
Residuals	85.0%	N/A	3.7%	6.9%	5.5%	**26.2%**

RSS: Relative sum of squares, tp1-3: predictive components, to: orthogonal component. The highest contribution for each component is reported in bold.

## Supplementary Materials

The following are available online at https://www.mdpi.com/2218-1989/10/7/269/s1, Table S1: Annotation and identification of metabolites with high effect-specific VIP^2^ value, according to the impact of the *Exposure* factor or to the *Exposure × Gender* interaction; Table S2: Two-way ANOVA tests in conjunction with Tukey’s multiple comparison test, followed by the Benjamini–Hochberg FRD correction.
